# Evaluation of perivascular fat attenuation with coronary CT angiography in cardiac transplantation patients: an imaging biomarker candidate for prediction of cardiac mortality and re-transplantation

**DOI:** 10.1007/s00330-023-09614-z

**Published:** 2023-04-19

**Authors:** Philipp T. Moser, Rüdiger Schernthaner, Christian Loewe, Andreas Strassl, Felix Denzinger, Sebastian Faby, Michael Wels, Volha Nizhnikava, Keziban Uyanik-Uenal, Andreas Zuckermann, Marie-Elisabeth Stelzmueller, Dietrich Beitzke

**Affiliations:** 1 Department of Biomedical Imaging and Image-Guided Therapy, Medical University of Vienna, Vienna, Austria; 2 Department of Radiology, Klinikum Landstrasse, Vienna, Austria; 3 Siemens Healthcare GmbH, Forchheim, Germany; 4 Department of Cardiac Surgery, Medical University of Vienna, Vienna, Austria

**Keywords:** Heart transplantation, Graft survival, Computed tomography angiography, Adipose tissue, Coronary artery disease

## Abstract

**Objectives:**

In cardiac transplant recipients, non-invasive allograft surveillance for identifying patients at risk for graft failure remains challenging. The fat attenuation index (FAI) of the perivascular adipose tissue in coronary computed tomography angiography (CCTA) predicts outcomes in coronary artery disease in non-transplanted hearts; however, it has not been evaluated in cardiac transplant patients.

**Methods:**

We followed 39 cardiac transplant patients with two or more CCTAs obtained between 2010 and 2021. We performed FAI measurements around the proximal 4 cm segments of the left anterior descending (LAD), right coronary artery (RCA), and left circumflex artery (LCx) using a previously validated methodology. The FAI was analyzed at a threshold of − 30 to − 190 Hounsfield units.

**Results:**

FAI measurements were completed in 113 CCTAs, obtained on two same-vendor CT models. Within each CCTA, the FAI values between coronary vessels were strongly correlated (RCA and LAD *R* = 0.67 (*p* < 0.0001), RCA and LCx *R* = 0.58 (*p* < 0.0001), LAD and LCx* R* = 0.67 (*p* < 0.0001)). The FAIs of each coronary vessel between the patient’s first and last CCTA completed at 120 kV were also correlated (RCA *R* = 0.73 (*p* < 0.0001), LAD *R* = 0.81 (*p* < 0.0001), LCx *R* = 0.55 (*p* = 0.0069). Finally, a high mean FAI value of all three coronary vessels at baseline (mean ≥  − 71 HU) was predictive of cardiac mortality or re-transplantation, however, not predictive of all cause-mortality.

**Conclusion:**

High baseline FAI values may identify a higher-risk cardiac transplant population; thus, FAI may support the implementation of CCTA in post-transplant surveillance.

**Key Point:**

• *Perivascular fat attenuation measured with coronary CT is feasible in cardiac transplant patients and may predict cardiac mortality or need for re-transplantation*.

## Background

Cardiac transplant recipients are at risk of cardiac death or need for re-transplantation due to graft failure [1]. After the first year of transplantation, cardiac allograft vasculopathy (CAV) becomes a leading cause of morbidity and mortality and affects 30% of patients within 5 years of transplant [1]. In CAV, endothelial dysfunction is followed by concentric intimal hyperplasia causing diffuse luminal narrowing of epicardial and intramyocardial coronary arteries and ischemic graft failure [2].

Acute graft rejection (ACR), either cellular or antibody-mediated, coronary artery disease, recurrence of the underlying myocardial condition, and cardiac malignancies are other, less prevalent causes of late cardiac mortality after transplantation [1]. Notably, many of the etiologies, including CAV [3], are associated with coronary artery inflammation and ultimately graft failure. This can occur with no or minimal symptoms because of autonomic denervation of the transplanted heart. Thus, transplant recipients routinely undergo post-transplant surveillance [4].

According to the most recent International Society for Heart and Lung Transplantation (ISHLT) consensus statement in 2010, invasive coronary angiography (ICA) is recommended for post-transplant CAV surveillance [5]. However, early disease may be underdiagnosed due to the diffuse and concentric character of lumen stenosis and the absence of a normal vessel caliber for reference [6]. Recent publications have suggested that evaluation of the coronary micro-circulation during ICA may be a valuable tool in predicting the development of ACR and CAV [7–9].

Since the last ISHLT consensus on CAV, technological advances in coronary CT Angiography (CCTA) of cardiac transplant recipients have led to increased use of non-invasive CCTA in routine post-transplant surveillance. CCTA detects severe CAV (> 50% lumen narrowing) with a sensitivity of 94%, specificity of 92%, negative predictive value of 99%, positive predictive value of 67%, and diagnostic accuracy of 94% [10]. In addition, CCTA provides more detailed anatomic information including coronary plaque characteristics, is safer, and is more cost-effective than ICA [11].

Recently, a novel imaging biomarker, fat attenuation index (FAI) of perivascular adipose tissue (PVAT), has garnered significant attention because of its prognostic value for adverse outcomes in non-cardiac transplant patients with coronary artery disease [12–15]. Coronary artery inflammation drives compositional changes of the PVAT surrounding the coronary arteries. In response to paracrine inflammatory stimuli released from the coronary vessel, PVAT becomes more aqueous and less lipophilic through inhibition of adipocyte differentiation and upregulation of lipolysis [16, 17]. These changes in PVAT associated with coronary artery inflammation can be detected as attenuation gradients (Hounsfield units, HU) on routine CCTA [18]. In the presence of increased inflammation, the measured FAI of PVAT increases. In this study, we aimed to assess the feasibility and prognostic value of FAI measurements with CCTA in a cardiac transplant cohort.

## Methods

### Study design

**Fig. 1:**
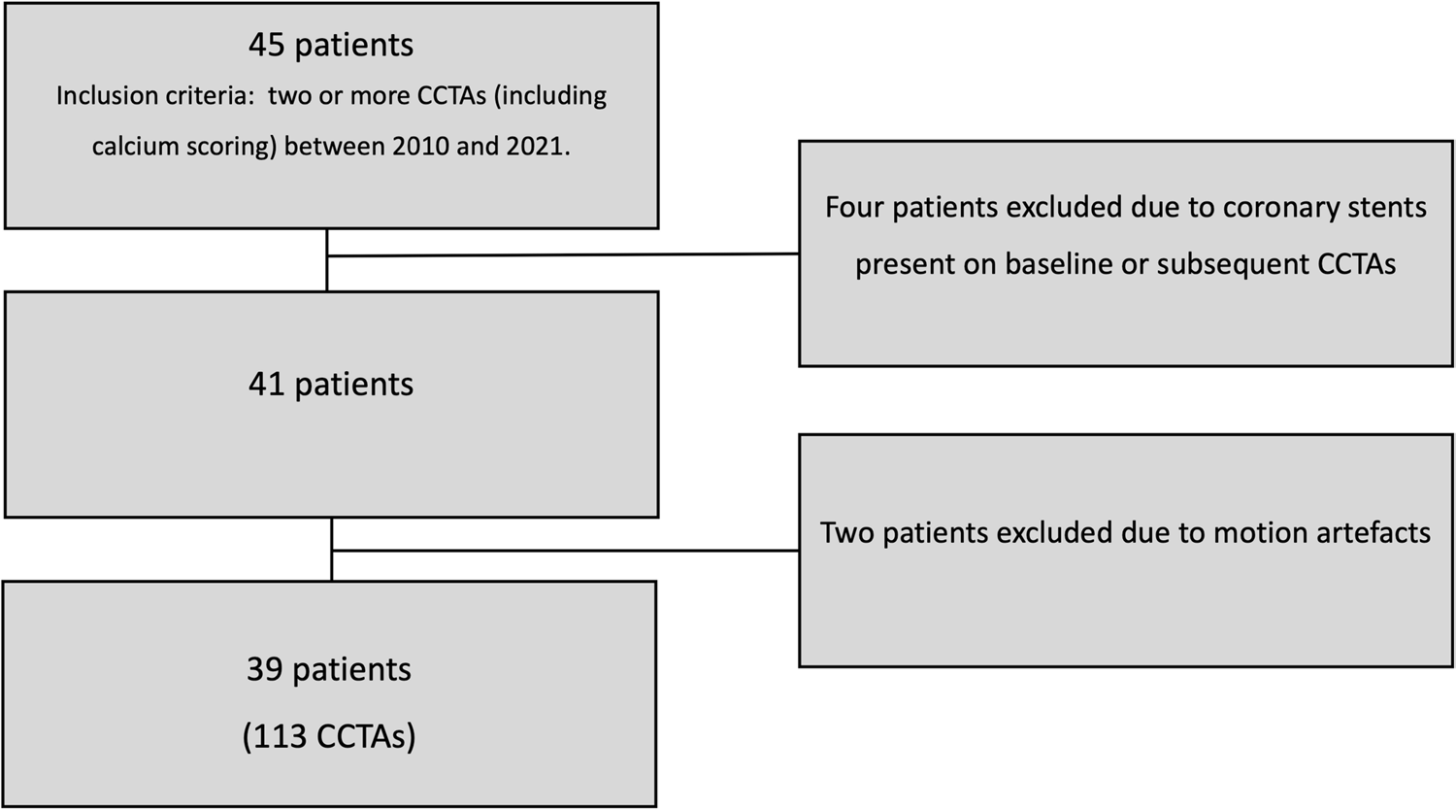
Study Design. Flowchart of patients from cardiac transplantation cohort included in the present analysis. CCTA: Coronary computed tomography angiography

We performed a longitudinal post hoc analysis of a cardiac transplantation patient cohort that received multiple routine post-transplant CCTAs at our hospital (Fig. 1). We included 45 sequential cardiac transplant patients, who underwent at least two coronary CCTAs (including calcium scoring) between 2010 and 2021. Four patients that received coronary artery stents (one patient prior to baseline CCTA, two patients prior to 2nd CCTA, and one patient prior to 3rd CCTA) were excluded. Additionally, two patients were excluded due to motion artefacts in the CCTA. Baseline characteristics were obtained from the electronic medical records, retrospectively. The research protocol was approved by the local institutional review boards and patient consent was waived due to the retrospective nature of the analysis.

### Cardiac CT and perivascular fat analysis

Electrocardiography (ECG)-gated CCTAs and non-enhanced cardiac CT for quantification of coronary artery calcium (Agatston score) were obtained according to clinically standardized protocols from two different dual-source CT (DSCT) machines from the same vendor (SOMATOM Definition Flash / SOMATOM Force, Siemens Healthineers). In Table 1 CT acquisition and reconstruction, parameters are presented in detail. ECG-gated CCTAs were performed with tube voltages ranging from 70 to 140 kV, depending on patient size. In total, 74 CCTAs (65%) were performed at 120 kV, 29 (26%) at 100 kV, and the remaining 10 (9%) at other kVs. Exams were performed using the “B26f” kernel and SAFIRE 3 iterative reconstruction on the DSCT SOMATOM Definition Flash or the “Bv36” kernel and ADMIRE 3 iterative reconstruction on the DSCT SOMATOM Force. The two convolution kernels have similar properties (The “B26f” kernel corresponds to the “Bv38” kernel in later versions) and iterative reconstruction was done at the same strength level.

**Table 1:** CT acquisition and reconstruction parameters

CT acquisition and reconstruction parameters	*n* (% miss.)	*n* (% overall)
CT system	113 (0%)	
DSCT Siemens SOMATOM Definition		102 (90%)
DSCT Siemens SOMATOM Force		11 (10%)
Mode		
Sequence		101
Helix		12
Tube voltage [kV]	113 (0%)	
100		29 (26%)
120		74 (65%)
Other		10 (9%)
Tube current [mAs]	113(0%)	
236 ± 68		
Collimation	113(0%)	
38.4		102 (90%)
50.4		6 (5%)
55.1		4 (4%)
40.8		1 (1%)
Slice thickness [mm]	113(0%)	
0.6		113 (100%)
Matrix Size		
512 × 512		113 (100%)
Kernel	113(0%)	
B26f		102 (90%)
Bv36		11 (10%)
Iterative Reconstruction	113(0%)	
SAFIRE 3		102 (90%)
ADMIRE 3		11 (10%)
FOV Size	113(0%)	
210 ± 17		

CT acquisition and reconstruction parameters. *CCTA*, coronary computer tomography angiography; *DSCT*, dual-source CT; *FOV*, field of view. Data presented as mean ± SD

**Fig. 2:**
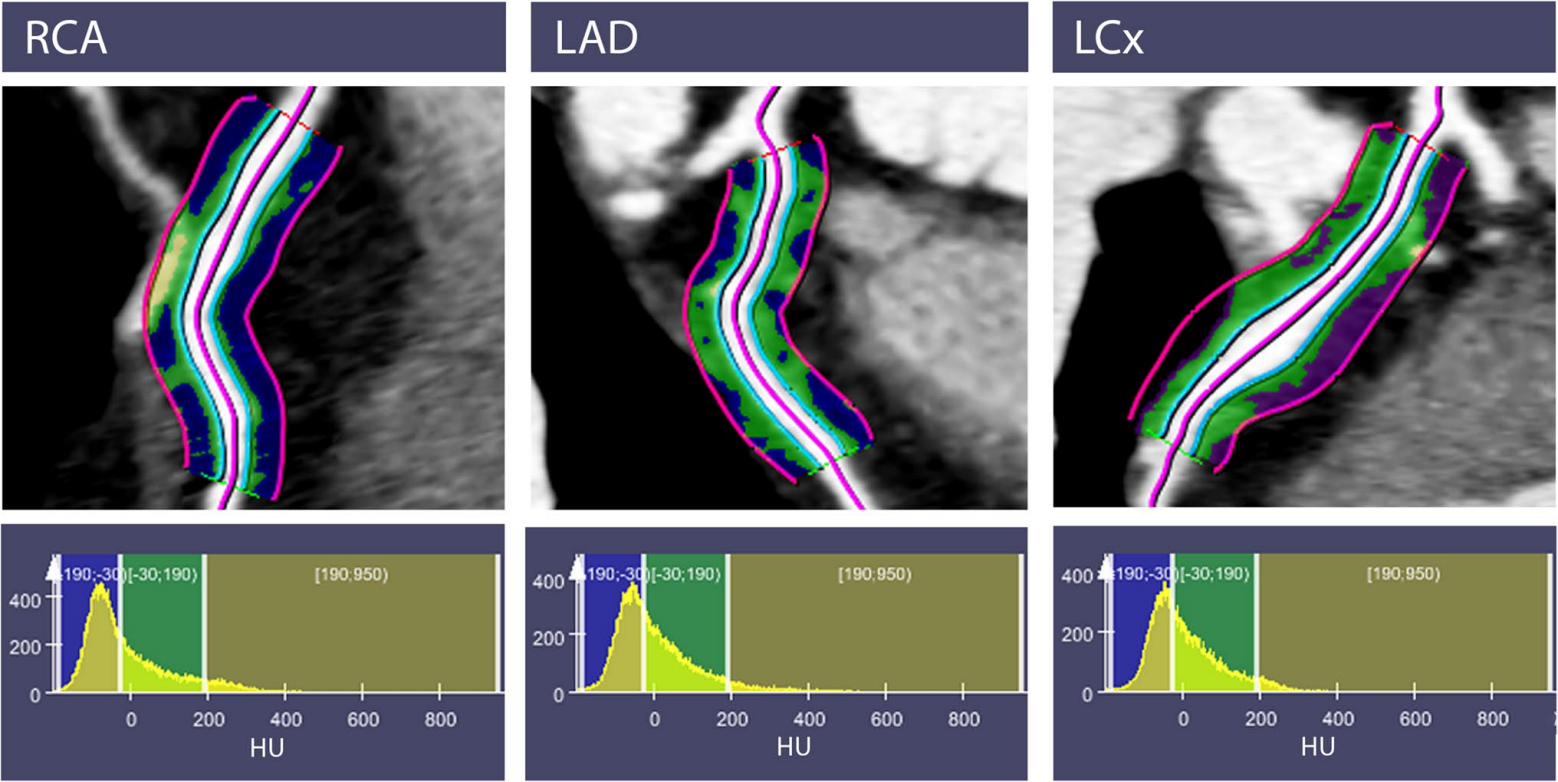
Assessment of the Fat Attenuation Index with CCTA. Top panel: Semi-automated detection of the PVAT surrounding the proximal RCA, LAD, and LCx. Bottom panel: Distribution of HU within the analyzed PVAT. PVAT, perivascular adipose tissue; RCA, right coronary artery; LAD, left anterior descending; LCx, left circumflex artery; HU, Hounsfield units

For the assessment of perivascular fat attenuation, we used reconstructed images with a slice thickness of 0.6 mm. We performed perivascular epicardial fat analysis (PVAT) in a semi-automated manner using a prototype post-processing and analytic software (Fig. 2, Coronary Plaque Analysis, syngo via Frontier, Siemens Healthineers) [19, 20]. PVAT was analyzed around the proximal left anterior descending (LAD), right coronary artery (RCA), and left circumflex artery (LCx) using a previously described and validated methodology [21]. In brief, PVAT volume was defined from the vessel wall radially at a length equal to the diameter of the vessel along 40 mm segments of the proximal LAD, LCx, and RCA. The analyzed RCA segment was 10 mm distal to its origin to avoid unwanted effects of the aortic root. PVAT within the volume of interest was defined as all voxels with attenuation between − 190 and − 30 HU. The FAI is the average CT attenuation of this PVAT. In total, the FAI could be obtained on 334/339 (98.5%) coronary PVAT measurements.

### Statistical analysis

All statistical analyses were performed in the R environment (The R Foundation for Statistical Computing, http://www.R-project.org) using R studio (version 2.1). For all analyses except for intra-CCTA comparisons and between tube voltage comparisons, only CCTAs performed at 120 kV were included. All baseline studies, which were used for outcome analysis, were performed with the same CT scanner model and scan protocols.

Continuous variables were reported as means (SD) or medians, [25th-75th] percentile, as appropriate, and count data as absolute frequencies (%). Normality was assessed using the Shapiro–Wilk test. Continuous variables between the two groups were compared using Student’s t-test and the Wilcoxon Rank Sum Test, as appropriate. Correlations between continuous variables were tested with Pearson’s r (including the coefficient of determination R). For some analyses, to normalize for differences in time between studies between patients, the change in FAI between studies was divided by the time (in days) between the studies. Finally, the association of perivascular FAI measurements with outcomes of interest was analyzed using the Cox proportional hazard models.

## Results

The study population consisted of 39 patients (80% men) who underwent cardiac transplantation. The median age at the time of transplant was 53 years old [25^th^-75^th^ percentile: 44–63], and the median age at the time of the first CTA was 65 years old [51–69]. In Table 2, patient demographics and co-morbidities are presented. Briefly, over a median follow-up time of 4.4 years [2.8, 5.0] from the first CCTA, patients returned for a total of 113 CCTAs obtained on two same-vendor CT models, with a mean number of 3 (SD ± 1) CCTAs per patient. The majority of CCTAs were performed with a tube voltage of 120 kV (*n* = 74, 65%); however, approximately one-quarter (*n* = 29, 26%) were performed using a tube voltage of 100 kV and the remaining 9 studies performed at other voltages. Over a median follow-up time of 18.0 years [13.2, 22.9] from transplantation to death, re-transplantation, or censorship, 8 patients died (21%) including 2 from acute cellular rejection, and one patient required re-transplantation due to severe CAV.

**Table 2:** Baseline characteristics of the study population

Variables	*n* (% miss.)	Overall
Demographics		
Male gender, *n* (%)	39 (0%)	31 (80%)
Hypertension	17 (56%)	11 (65%)
Hyperlipidemia	18 (54%)	11 (61%)
Type 2 diabetes mellitus	17 (56%)	5 (29%)
Cyclosporine	38 (3%)	18 (47%)
Sirolimus	38 (3%)	20 (26%)
Tacrolimus	38 (3%)	12 (32%)
Everolimus	38 (3%)	1 (3%)
Two or more immunosuppressants	38 (3%)	3 (8%)
Age at transplant, years [IQR]	39 (0%)	53 [44,63]
Number of CCTAs, mean (SD)	39 (0%)	3 (1)
Time from transplant to 1st CCTA, years [IQR]	39 (0%)	8 [5,12]
Time from transplant to last CCTA, years [IQR]	39 (0%)	12 [8,16]
Coronary artery calcium at first study	39 (0%)	13 (33%)

Baseline characteristics of the study population (*n* = 39). *n* (%miss.) reports the number of patients with fully observed data (% missing). *IQR*, interquartile range (median [25^th^–75^th^] percentile)

**Fig. 3:**
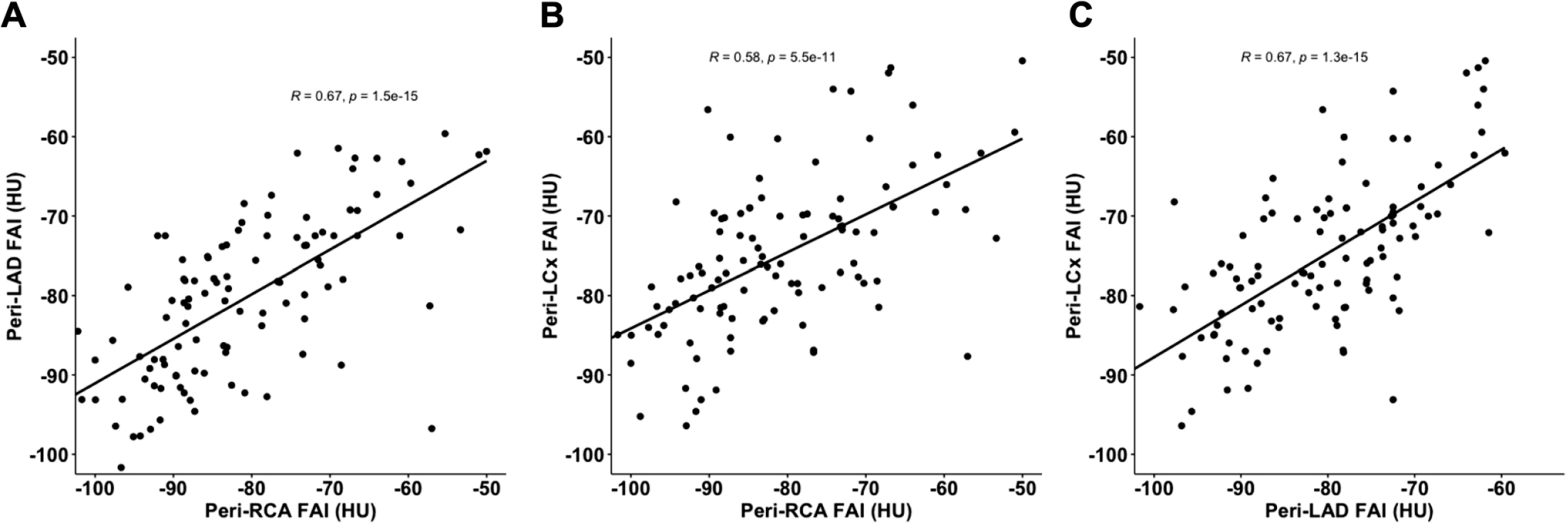
Bivariate association of FAI measured around the three major coronary vessels within CCTA. Correlation between FAI values measured around RCA, LAD, and LCx in all CCTAs (*n* = 113). **A** Correlation between RCA and LAD. **B** Correlation between RCA and LCx. **C** Correlation between LAD and LCx. FAI, fat attenuation index of the perivascular adipose tissue; HU, Hounsfield Units; LAD, left anterior descending artery; LCx, left circumflex; RCA, right coronary artery

Within 113 CCTA, the FAI between coronary vessels was strongly associated. In detail, the correlation coefficient between the RCA and LAD was 0.67 (*p* < 0.0001), between RCA and LCx was 0.58 (*p* < 0.0001), and between the LAD and LCx was 0.67 (*p* < 0.0001) (Fig. 3). As expected, the FAI values of each coronary vessel between studies measured at 120 kV versus non-120 kV were significantly different (RCA − 77.9 ± 12.2 vs. − 88.0 ± 9.2, *p* < 0.0001; LAD − 77.5 ± 9.1 vs − 86.2 ± 9.4, *p* < 0.0001; LCx − 72.6 ± 10.2 vs − 79.6 ± 7.3, *p* < 0.0001, respectively).

Given the effect of kV on FAIs, for all subsequent analyses we included only studies conducted at 120 kV (*n* = 74). Of note, 38 of the 39 patients had the baseline CCTA performed at 120 kV. The perivascular FAI of each coronary artery was normally distributed for all studies (RCA − 77.9 ± 12.2, LAD − 77.5 ± 9.1, LCx − 72.6 ± 10.2) and there were no significant differences between the perivascular fat attenuation measurements obtained at 120 kV on either CT machine (RCA *p* = 0.38, LAD *p* = 0.14, LCx *p* = 0.68).

We then explored whether patient characteristics were associated with the baseline perivascular FAI measurements (*n* = 38 patients). Females had significantly higher baseline RCA and LAD FAI measurements compared to males (mean − 67.5 ± 3.8 vs. − 80.8 ± 10.8 *p* < 0.0001, mean − 70.6 ± 4.5 vs. 78.0 ± 8.2 *p* = 0.0027, respectively). Additionally, baseline LAD FAI measurements for patients with hypertension were lower than those without (mean − 81.2 ± 8.6 vs − 70.0 ± 3.9, *p* = 0.0023). Age at transplant, time to first cardiac CTA, hyperlipidemia, and type of immunosuppression were not associated with any of the baseline FAI measurements.

**Fig. 4:**
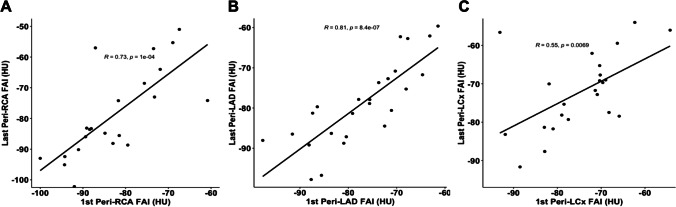
Bivariate association of a patient’s FAI measured around the three major coronary vessels between CCTAs at 120 kV. Correlation between FAI values measured around RCA, LAD, and LCx between patient’s 1^st^ and last CCTA taken at 120 kV. **A** Measurements of peri-RCA FAI in patient’s first and last CCTA. **B** Measurements of peri-LAD FAI in patient’s first and last CCTA. **C** Measurements of peri-LCx FAI in patient’s first and last CCTA. FAI, fat attenuation index of the perivascular adipose tissue; HU, Hounsfield units; LAD, left anterior descending artery; LCx, left circumflex; RCA, right coronary artery

We then sought to assess the relationship between a patient’s perivascular FAI measurements over time. In this analysis, we included the 25 patients with a baseline CCTA and at least one follow-up CCTA conducted at 120 kV. The mean time between these patients’ baseline CCTA and their last CCTA conducted at 120 kV was 2.8 years (± 2.3 years). We found that the FAI measurements of each vessel in the patient’s first and last study were highly correlated. The correlation coefficient between the FAI of the RCA in the first and last study was 0.73 (*p* < 0.0001), of the LAD was 0.81 (*p* < 0.0001), and of the LCx was 0.55 (*p* = 0.0069) (Fig. 4).

Given the increased risk of cardiac mortality in heart transplant patients, we evaluated whether baseline perivascular fat attenuation measurements of each coronary vessel were predictive of all-cause mortality or cardiac mortality and need for re-transplantation. Notably, FAI values in the perivascular fat of each coronary vessel were associated with cardiac mortality or re-transplantation, however, were not associated with all cause-mortality (Table 3).

**Table 3:** Association of perivascular FAI around the RCA, LAD, and LCx and all-cause and cardiac mortality or cardiac re-transplantation

	All-cause mortality		Cardiac mortality or cardiac re-transplantation	
	Adjusted HR (95% CI)	*p* value	Adjusted HR (95% CI)	*p* value
RCA	1.00 (0.93–1.08)	0.99	**1.15 (1.02–1.30)**	**0.021**
LAD	1.02 (0.93–1.12)	0.74	**1.41 (1.06–1.87)**	**0.018**
LCx	0.99 (0.92–1.08)	0.96	**1.17 (1.02–1.34)**	**0.027**

Association of the FAI of RCA, LAD, and LCx with all-cause mortality  and cardiac mortality and or need for re-transplantation.  Data presented as adjusted Hazard Ratio (HR) with a 95% confidence interval (CI).  *RCA*, right coronary artery; *LAD*, left anterior descending artery; *LCx*, left circumflex artery

Baseline FAI measurements of the RCA, LAD, or LCx were not associated with the subsequent development of coronary calcifications. Furthermore, the change in the FAI measurement between the 1st and 2nd CCTA for each vessel (normalized for the time between studies) was not significantly different between patients who did or did not subsequently become Agatston positive (median duration of follow-up after 2nd is 1.7 years [1.4, 3.2]).

**Fig. 5:**
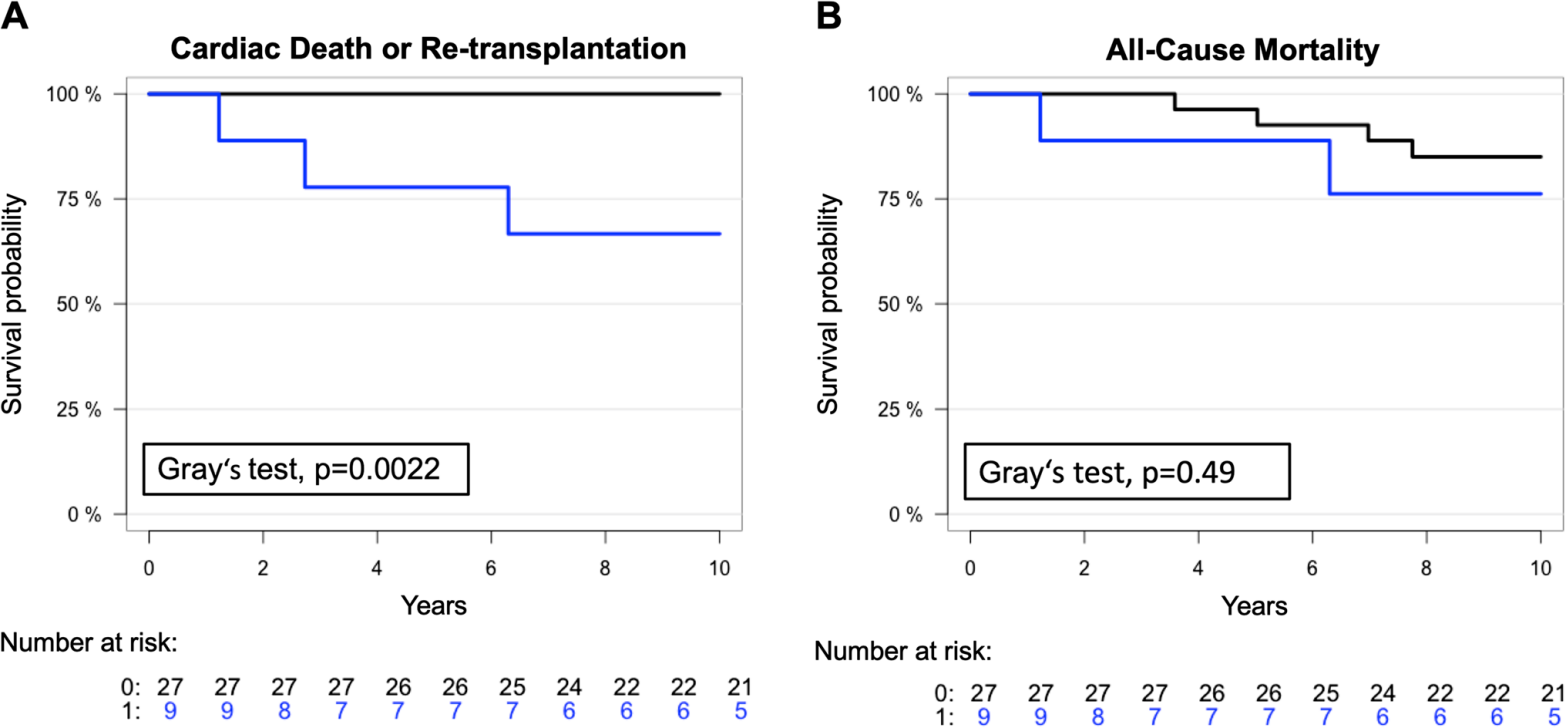
Kaplan–Meier curves of cardiac mortality and all-cause mortality with high versus low mean baseline perivascular FAI. Patients were dichotomized into high versus low perivascular fat attenuation groups based on average FAI in the baseline CCTA. High-risk patients (blue line) were defined by mean perivascular FAI ≥  − 71 HU and low-risk patients (black line) by mean perivascular FAI < -71 HU. Mortality curves show the risk of cardiac death or need for re-transplantation (**A**) and all-cause mortality (**B**). Differences in mortality incidence functions between groups were investigated using Gray’s test

Based on these observations and to explore whether baseline FAI values could be used to stratify a patient’s risk of all-cause or cardiac-specific mortality, patients were dichotomized into high versus low perivascular fat attenuation groups based on average FAI in the baseline CCTA. The threshold was set at − 71 HU, which was defined by the value of the highest quartile. We found that patients with a high mean baseline FAI were at higher risk for cardiac death or re-transplantation, with a 10-year cumulative risk of death of 33% [95%CI:] vs. 0% [95%CI:] for patients in the lower baseline FAI group (Gray’s test, *p* = 0.0022, Fig. 5A). However, there was no difference between these groups for all-cause mortality, with a 10-year cumulative risk of death of 24% [95%CI:] vs. 15% [95%CI:], respectively (Gray’s test, *p* = 0.49, Fig. 5B).

## Discussion

CCTA has demonstrated diagnostic value in post-transplant surveillance and is more frequently used in routine clinical care [10]. The FAI of PVAT surrounding coronary arteries measured with CCTA is a promising quantitative, reader-independent imaging biomarker for coronary artery inflammation [21]. The diagnostic and prognostic value of the FAI for cardiac allograft disease has not yet been studied. Here, we evaluated the FAI in a cardiac transplantation cohort of 39 patients with 113 CCTA exams, showing that baseline FAI values of each coronary artery were predictive of cardiac mortality or need for re-transplantation.

Measurements of the FAI of PVAT with CCTA can be done without an additional radiation dose or patient examination time. We show that FAI measurements are feasible in CCTAs of cardiac transplant patients, a patient population with an increased heart rate due to cardiac denervation post-transplantation and frequent foreign material that may lead to imaging artefacts.

While the FAI values from CCTAs obtained at 120 kV from two same vendor machines did not differ significantly, tube voltage had a significant influence on FAI values. This is consistent with findings by Ma et al., showing also differences in FAI assessment based on different kV settings in a non-transplant cohort [22]. In our cohort, due to long follow-ups between the first and last CT (median 4.4 years), for many patients, the tube voltage changed between CCTAs. However, for the best inter- and intra-patient comparison, the same kV is recommended. The challenges due to variations in tube voltage may be addressed with the use of novel photon-counting CT and virtual monoenergetic image reconstruction in routine clinical practice [23].

We assessed the perivascular fat attenuation in proximal coronary arteries surrounded by epicardial fat through the FAI. The FAI of coronary arteries within an individual was highly correlated. Detected high values may, therefore, reflect a global inflammatory state of the coronary artery tree such as is present in CAV [24] and, thus, measuring the FAI of one reference coronary artery segment may be sufficient. To date, little is known about the dynamic of the FAI values over time measured in serial CCTAs. We found a strong correlation between a patient’s FAI over time with a mean increase in the FAI of the RCA and LCx, which may represent progressive coronary artery inflammation. There are a number of hypotheses that could explain the observed correlation between a patient’s FAI at the first and last exam. The development of coronary inflammation post-transplant occurs before 8 years, the median time from transplant to first CCTA in this study. Additionally, the observation time between the CCTAs may have been too short to see changes in FAI, or patients who would have had significant changes in their FAI measurements on their second CCTA did not survive this study.

The changes in a patient’s FAI measurements over time were not correlated to the time between studies. This may be explained by CCTAs being ordered more frequently in patients deemed at higher risk for post-transplant complications.

We observed significantly higher baseline FAI measurements in females, which is inconsistent with studies on native hearts [21]. This may be due to underlying differences in immune response between genders or alternatively due to a bias in our population due to the retrospective nature.

In our cohort, different immune suppressant agents were not associated with significant differences in the FAI. Previously, it has been shown that in native hearts the FAI can be altered through disease-modifying therapies. Biologic treatments, but not non-biologic treatments, resulted in a significant reduction of PVAT FAI within 12 months from treatment start in patients suffering from psoriasis [25]. Whether the FAI could be used as an imaging biomarker for adequate immunosuppression needs further assessment in cardiac transplant patients.

Evaluation of calcium deposition by Agatston scoring of native and transplanted hearts is a well-defined predictor of cardiac death [26, 27]. While coronary calcium deposition is associated with a long-standing inflammation, we did not observe a correlation with FAI and positive Agatston Score in the baseline studies, which were completed with a median of 9.5 years [4.8, 12.2] after cardiac transplantation. This is consistent with the results of the large CRISP-CT study in non-transplant patients showing that an elevated Calcium Score is not associated with high FAI values [21]. In addition, we did not find a correlation between FAI at baseline or change in FAI over time with the development of de-novo coronary calcium deposition. In the coronary arteries of transplanted hearts, calcification is found in traditional atheroma and in severe CAV. In our cohort, only one patient had severe CAV at baseline and no patients developed de-novo CAV during observation. This patient had high FAI baseline values (RCA − 50.0; LAD − 61.9 HU; − 50,4 HU LCx) and an Agatston score of 1. Therefore, the lack of association between FAI and coronary calcification does not suggest that FAI may not have value in predicting CAV.

In our patient cohort the mean FAI of the LAD, RCA, and LCx in the baseline CCTA exam was predictive of cardiac mortality or need for re-transplantation, however, did not predict all cause-mortality. In detail, in our cohort, two patients died from acute cellular rejection, and a third required re-transplantation due to severe CAV. The mean baseline FAI values of these patients were − 64.2, − 67.4, and − 54.1, respectively, all of which were in the highest quartile of mean baseline FAI measurements. The patients’ baseline CCTA studies were obtained at 1.2, 6.3, and 2.7 years, respectively, before death or re-transplantation.

The FAI imaging biomarker may also be valuable in risk stratification of coronary artery disease, which is often concomitant with CAV. In native hearts, changes in FAI can distinguish between unstable and stable coronary artery plaques in patients with acute coronary syndrome [18]. In cardiac allografts, this would be additional information not provided by ICA post-transplant surveillance.

Our study has several limitations. First, given the retrospective design and the inclusion via an imaging modality, we have limited information about baseline characteristics including complete information on immunosuppressive therapies. Given the known effect of tube voltage on FAI, we only included CCTAs performed at 120 kV for most analyses. The generalizability of our threshold for a high mean FAI at baseline would have to be validated in CCTAs performed at other kVs. Additionally, due to the small number in our study, we were unable to control for traditional cardiovascular risk factors in our assessment of baseline FAI measurements and future cardiac death. Finally, our cohort likely has a bias towards a healthier transplant population to be included patients needed to survive to first CCTA, and patients with coronary stents were excluded.

## Conclusion

The FAI of PVAT is a novel non-invasive, quantitative imaging biomarker assessed with conventional CCTA. In our cardiac transplant follow-up cohort, FAI values of coronary arteries within a CCTA and FAI values of coronary arteries in a patient’s serial CCTAs, performed at 120 kV, were highly correlated. Additionally, baseline FAI values of each coronary artery were predictive of cardiac mortality or need for re-transplantation. These findings suggest that even a single FAI measurement of coronary vessels post-transplantation may identify a higher risk cardiac transplant population. A prospective study with predefined imaging parameters and timing would be valuable in further assessing whether incorporation of FAI measurements into CCTA post-transplant surveillance can be used for risk stratifying patients, may facilitate identification of patients who would benefit from more aggressive therapies, and the optimal timing of these measurements.

## Abbreviations

ACR: Acute graft rejection
CAV: Cardiac allograft vasculopathy
CCTA: Coronary CT angiography
DSCT: Dual-source CT
ECG: Electrocardiography
FAI: Fat attenuation index
HU: Hounsfield units
ICA: Invasive coronary angiography
IQR: Interquartile range
ISHLT: International Society for Heart and Lung Transplantation
LAD: Left anterior descending
LCx: Left circumflex artery
PVAT: Perivascular adipose tissue
RCA: Right coronary artery

## Declarations

### Guarantor

The scientific guarantor of this publication is Philipp T Moser.

### Statistics and biometry

No complex statistical methods were necessary for this paper.

### Methodology

• Retrospective

• Diagnostic or prognostic study / observational

• Performed at one institution
